# Local patterns of arbuscular mycorrhizal fungal diversity and community structure in a natural *Toona ciliata* var. *pubescens* forest in South Central China

**DOI:** 10.7717/peerj.11331

**Published:** 2021-05-03

**Authors:** Jianfeng Pan, Qiong Wang, Xiaoyan Guo, Xueru Jiang, Qiangqiang Cheng, Li Fu, Wei Liu, Lu Zhang

**Affiliations:** 1 Jiangxi Provincial Key Laboratory of Silviculture/Collaborative Innovation Center of Jiangxi Typical Trees Cultivation and Utilization, Nanchang, Jiangxi, China; 2 College of Forestry/College of Art and Landscape, Jiangxi Agricultural University, Nanchang, Jiangxi, China; 3 Nature Protected Area Construction Center of Jiangxi, Nanchang, Jiangxi, China

**Keywords:** Arbuscular mycorrhizal fungi, *Toona ciliata* var. *pubescens*, Natural regeneration

## Abstract

*Toona ciliata* var. *pubescens* (*Toona* in Meliaceae) (*Tc*) is listed as an endangered species, and there are natural regeneration obstacles due to its long-term excessive exploitation and utilization. Arbuscular mycorrhizal fungi (AMF) can produce beneficial effects for plant growth and natural regeneration. However, the characteristics of the AMF community in natural *Tc* forests are poorly understood. The Illumina PE250 high-throughput sequencing method was used to study the characteristics of the AMF community in the rhizosphere soil and roots associated with three dominant tree species (*Tc*; *Padus buergeriana*, *Pb*; and *Maesa japonica*, *Mj*) in a natural *Tc* forest in Guanshan National Natural Reserve, South Central China. The results found that Glomeraceae was the most abundant AMF family in the rhizosphere soil and roots. Moreover, the relative abundance of Archaeosporaceae in rhizosphere soil was significantly larger than that in the roots; in contrast, the relative abundance of Glomeraceae in rhizosphere soil was significantly lower than that in the roots (*p* < 0.05). Regarding different tree species, the relative abundances of Acaulosporaceae and Geosiphonaceae were larger in *Mj* and *Tc* than in *Pb*. AMF operational taxonomic units (OTUs) were 1.30-, 1.43-, and 1.71-fold higher in the *Tc*, *Pb*, and *Mj* rhizosphere soil, respectively, than in the corresponding roots. Nevertheless, higher AMF community richness was found in the roots compared to that in the rhizosphere soil based on the Chao index. This finding indicated that AMF of a relatively high aggregation degree were in roots, and more AMF groups with relatively low abundance occurred in the rhizosphere soil, which correspondingly lowered the calculated richness index of the AMF community. A redundancy analysis showed that different soil chemical properties impacted variations in the AMF community characteristics differently. This study has great significance for the interpretation of AMF diversity survey and the application design of AMF in vegetation restoration.

## Introduction

*Toona ciliata* var. *pubescens* (*Tc*), which is listed as an endangered species and national secondary protected plant, is a precious wood plant of the *Toona* genus in the Meliaceae family with high economic value and development prospects (Liu, Jiang & Chen, 2014). Due to long-term excessive exploitation and utilization, large changes in habitat, and obstacles to natural regeneration, the existing natural *Tc* forests are limited (Zhan et al., 2019). The distribution area of *Tc* is largely in habitats such as valleys and streams, the areas of which are also gradually declining. Therefore, how to protect and increase *Tc* populations has become an urgent issue to be addressed. In recent years, some researchers have attentioned to the community structure of *Tc* forests (Fu et al., 2007) and their population dynamics (Huang et al., 2013), spatial genetic structure and genetic diversity (Liu et al., 2013) and regeneration status (Huang et al., 2018). However, there are relatively few studies on the obstacles to natural regeneration of *Tc* forests, and the obstacles are the greatest threat to endangered natural *Tc* forests. Seeds of *Tc* are small and light, and the fruiting of this species exhibits unstable yields (Zhang, Zhang & Lv, 2008). The low availability of seeds leads to a low effective transmission, which causes provenance restriction; seeds cannot spread to suitable sites and are not easily transmitted (Münzbergová & Herben, 2005). After spreading to a microhabitat, plants are unable to grow into young trees due to the influence of biological and abiotic factors, resulting in restrictions on population regeneration (Marques & Burslem, 2015). Regarding *Tc*, the heterogeneity of its microhabitat may be the main reason for its limited natural regeneration (Guo et al., 2017). Microhabitat ecological factors include a variety of limiting factors, such as soil, water, light, and soil microorganisms. These factors determine the formation of young trees, and there are differences in the dominant factors of different species of plants. Soil microorganisms play vital roles in seedling growth and natural regeneration in *Tc* (Guo et al., 2017). Huang, Zhang & Liao (2012) indicated that pathogen infection was the main cause of seed rot and natural regeneration failure of *Tc*. Arbuscular mycorrhizal fungi (AMF), which have a symbiotic association with over 80% terrestrial plants (Smith & Read, 2008), are beneficial to plant growth and undoubtedly play important roles in the natural regeneration of *Tc* (Dhar & Mridha, 2012; Thapar, Vijyan & Uniyal, 1992).

AMF are widely distributed in soil ecosystems, and their growth and reproduction are strongly linked to the soil environment and host plant characteristics since fungi generally enhance stress tolerance, plant growth, and nutrient uptake (Jeffries et al., 2003). AMF have important ecological significance in the succession and stability of plant community structure (Yang et al., 2014). At the ecosystem scale, AMF increase the absorption capacity of plants to insoluble nutrients (such as Zn, P, and Ca) through the expansion of external hyphae and the secretion of organic acids; AMF can also improve the resistance of plants to external adverse environments, diseases and insect pests through site occupation and antagonism (Smith & Read, 2008) to increase ecosystem stability (e.g., secrete glomalin to form aggregates for increasing soil structural stability) (Caravaca et al., 2005) and change the succession direction of plant communities (Smith & Read, 2008). At the individual plant scale, the symbiotes formed by AMF and plants can improve the stomatal conductance and transpiration rate of plant leaves and increase the net photosynthetic rate and chlorophyll content of plants (Hoeksema et al., 2010). Thus, AMF promote the growth of host plants, increase plant biomass (Yang et al., 2014), and improve the competitiveness of plants in natural habitats. The light conditions of natural *Tc* forests are poor, and the *Tc* seedlings are hindered at the initial growth stage (Huang, Zhang & Liao, 2012). AMF can participate in plant carbon assimilation and metabolism by increasing the chlorophyll content in plant leaves and enhancing photosynthesis, thus promoting plant growth and development (Liu & Li, 2000). *Tc* is mainly distributed in Jiangxi, Hunan, Hubei, Guangdong, Fujian, Zhejiang and Yunnan provinces in South Central China, most of which have red soil. Red soil is an acidic ferrous bauxite with low base saturation, and it is acidic, tacky and nutrient deficient (especially of phosphorus [P]) (Sun, Zhang & Zhao, 1995). These soil conditions may hinder the natural regeneration of *Tc* seedlings. The hyphal network formed by AMF can secrete organic acids and increase the spatial availability of insoluble nutrients (especially P) and their long-distance transport capacity, which can provide more nutrients for host plants and promote plant growth (Feng & Li, 2001). Therefore, AMF might affect the natural regeneration process of *Tc* by improving its P nutrition status. The mycelial and Glomalin-related soil proteins produced by AMF can also promote soil aggregate formation, thus providing a good soil environment for plant root growth (Gillespie et al., 2011) and improving plant resistance to soil-borne diseases and drought (Ji, Tan & Chen, 2019). In a previous study by our group, pathogens were the main cause of seed rot and natural regeneration failure of *Tc* (Huang, Zhang & Liao, 2012). AMF also have a certain antagonistic effect on soil-borne diseases, which plays an important role in the germination of plant seedlings (Kumar, Sharma & Mishra, 2010). At present, there are few studies on AMF and the natural regeneration of *Tc* (Dhar & Mridha, 2012; Thapar, Vijyan & Uniyal, 1992). Therefore, from the perspective of soil microorganisms, especially AMF, exploring the internal relationship between microorganisms and strategies for natural regeneration of endangered *Tc* can provide a unique perspective to understand the endangerment mechanism and survival strategy of *Tc*.

Illumina PE250 high-throughput sequencing method was used to investigate the characteristics of the AMF community in three dominant tree species in a natural *Tc* forest in this study. We hypothesized that there would be differences in the AMF community composition and diversity among the three root species, among the three soil types and between root and soil in dominant tree species and that the changes in tree species and soil chemical properties may explain these variations. These results are expected to provide avenues for the protection and cultivation of *Tc* forests in terms of AMF investigation.

## Materials & Methods

### Study area description

**Figure 1:**
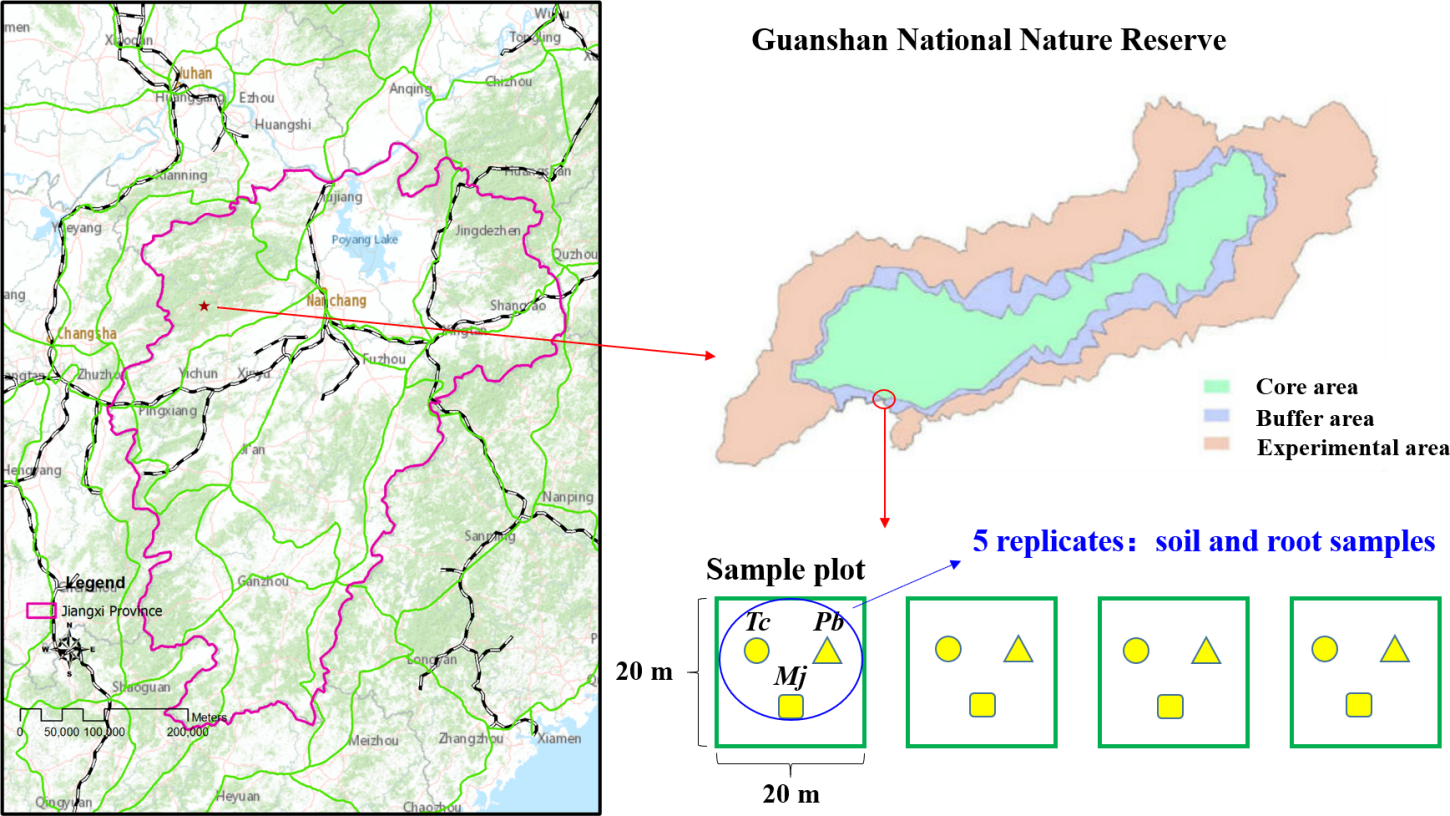
Location of Guanshan National Nature Reserve and study sites.

The study site (114°29′−114°45 ′E, 28°30′−28°40′N) is located in the Guanshan National Nature Reserve, Jiangxi Province, China (Fig. 1). The vegetation type of the study site is that of a subtropical evergreen broad-leaved forest. The climate is warm and humid, with four distinct seasons. The mean annual temperature and precipitation are 17.2 °C and 1,680.2 mm, respectively. Wild plant resources are abundant in Guanshan; there are 2,344 species of higher plants, and some rare plants, such as *Ginkgo biloba* L., *Taxus wallichiana* var. mairei, *Bretschneidera sinensis* Hemsl., *Amentotaxus argotaenia* (Hance) Pilger, *Halesia macgregorii* Chun, *Disanthus cercidifolius* Maxim. var. *longipes* Chang, *Emmenopterys henryi* Oliv., *Glycine soja Sieb. et* Zucc., *Ormosia henryi* Prain, *Magnolia officinalis subsp.biloba* (Rehd. et Wils.) Cheng et Law, *Phoebe bournei* (Hemsl.) Yang, and *Tc*, are mainly distributed in Guanshan Nature Reserve. Guanshan is regarded as one of the best experimental sites for studying forest ecosystems (Yao et al., 2017). Typical red soil is found at this study site.

### Experimental design and sampling

*Tc*, which is listed as an endangered species and a national secondary protected plant, is a precious wood plant of the *Toona* genus in the Meliaceae family. The community of natural *Tc* forests is mostly located in the subtropical evergreen broad-leaved forest, and the arbor layer is dominated by heliophilous tree species. A field investigation was conducted in October 2016, and four experimental plots of 20 m ×20 m were established in the natural *Tc* forest (Fig. 1). According to the dominance characteristics of plants, *Tc*, *Maesa japonica* (*Mj*), and *Padus buergeriana* (*Pb*) were the dominant plants in the four experimental plots. Rhizosphere soil and root samples were collected from five random individuals of each tree species. A sterile stainless-steel spatula was used to excavate plant roots in four different directions, and roots and rhizosphere soil in four directions were mixed as one sample. Finally, 15 rhizosphere soil samples and 15 root samples per plot (three tree species) were obtained. Then, each rhizosphere soil sample was mixed and homogenized by passing through a <2-mm sieve to remove aboveground plant materials, roots and stones and then divided into two parts. One subsample was stored at −80 °C for DNA extraction, and the other was air-dried, gently ground, and passed through a 0.149-mm mesh sieve for future chemical analyses. Root samples were cleaned with sterile water and stored in a −80 °C freezer for DNA extraction.

### Soil chemical property determination

The soil pH value was determined by the glass electrode method (water to soil ratio, v:w = 2:1). Soil organic matter (SOM) was determined by the potassium dichromate method with external heating. Total nitrogen (TN) was determined by the Kjeldahl method. Total phosphorus (TP) was determined by sulfuric acid-perchloric acid elimination and the molybdenum-antimony colorimetric method. Available phosphorus (AP) was determined by 0.5 M NaHCO_3_ extraction colorimetry. Total potassium (TK) was extracted with a 1 M NH_4_OAc solution (pH = 7.0) and determined by flame photometry. These determinations were conducted according to Bao (2000).

### DNA extraction

A FastDNA SPIN Kit (MP Biomedicals LLC, Santa Ana, CA) was used to extract soil DNA in accordance with the operation manual (0.5 g of fresh soil sample). A Fast Plant Kit (Beijing Tiangen) was used to extract root DNA in accordance with the operation manual (0.05 g of frozen root samples). The total DNA concentration of the extracted root/soil DNA in each root or soil sample was measured using NanoDrop ND-8000 (NanoDrop, Wilmington, DE) spectrophotometry. The extracted DNA was diluted to 10–20 ng µL ^−1^ with ultra-pure H_2_O and stored at −20 °C.

### PCR amplification

To ensure the accuracy and reliability of subsequent data analysis, PCR reaction should meet two conditions: (1) Using the low cycle number in the process of amplification as far as possible; (2) Ensuring the same number of cycles for each sample amplification. The primers were AMV4.5NF-F 5′-Barcode-AAGCTCGTAGTTGAATTTCG-3′ and AMDGR-R 5′-CCCAACTATCCCTATTAATCA-3′. The PCR reaction system (20 µL volume) contained 2 µL ddH_2_O, 10 µL template DNA, 0.4 µL FastPfu Polymerase, 0.8 µL Reverse Primer (5 µM), 0.8 µL Forward Primer (5 µM), 2 µL 2.5 mM dNTPs, and 4 µL 5 × FastPfu Buffer (four replicates per sample).

The PCR reaction system was gently mixed and placed in a PCR instrument (ABI GeneAmp^®^ type 9700). The regime used was as follows: 95 °C for 5 min; 95 °C for 30 s, 55 °C for 30 s, 72 °C for 45 s; 27 cycles of 95 °C for 30 s, 55 °C for 30 s, 72 °C for 45 s, followed by 72 °C for 10 min. The PCR products were separated by 2% agarose gel (120 V for 40 min) and purified with an extraction kit (Aidlab Biotechnologies Co., Ltd) according to the operation manual. The concentration of purified products was quantified spectrophotometrically using a TBS-380.

### Illumina high-throughput sequencing and bioinformatics

Purified PCR products were sequenced by Illumina PE250 High-throughput sequencing platform (Shanghai BIOZERON Co., Ltd) according to standard protocols. Sequences were grouped into operational taxonomic units (OTUs) with a 97% identity threshold (Mahmoudi et al., 2019). The most abundant sequence from each OTU was selected as a typical sequence for the OTU. The cluster of sequences was performed by Usearch (version7.1 http://drive5.com/uparse/) (Edgar, 2013). Taxonomy was assigned to fungal OTUs against a subset of the Silva 104 database (http://www.arb-silva.de/download/archive/qiime/). The typical sequences were further confirmed by Genbank (http://www.ncbi.nlm.nih.gov/) for the OTUs that could not be identified at the level of families or classes in the above fungal database. The raw sequences described here are accessible via GenBank with SRP277481 and accession numbers SAMN15815414 to SAMN15815425, SAMN15815492 to SAMN15815503.

### Diversity and richness indexes

The Chao index was used to estimate the total number of species in the ecosystem (Chao, 1984). Chao 1 was used to estimate the number of OTUs. The Simpson (Simpson, 1949) and Shannon (Algoet & Cover, 1988) were used to quantitatively estimate microbial diversity.

The coverage index was also determined. The higher the coverage of each sample library was, the larger the probability that the sequence is detected in a sample, while relatively low coverage indicates a low probability of detection. This index reflects whether the sequencing results represent the true state of the microbes in the sample.

\begin{eqnarray*}C=1- \frac{{n}_{1}}{N} \end{eqnarray*}

where *C* is the coverage of OTUs, *n*_1_ is the number of OTUs that contain only one sequence (singletons), and *N* is the total sequence.

### Data analysis

Soil chemical properties, AMF community composition and diversity in different niches (roots and rhizosphere soil) among tree species were subjected to analysis of variance and then the means were compared using Duncan’s multiple range test at *p* <0.05, by the SPSS 22.0 software (IBM, Armonk, NY, USA). Redundancy analysis (RDA) was used to explore associations between soil chemical properties and the characteristics of the AMF community in the roots and rhizosphere soil (Canoco 5.0, Biometrics, Netherlands).

## Results

### Soil chemical properties

The average soil pH value, SOM, TN, TP, AP, and TK contents were 4.51, 127.92 g kg^−1^, 5.92 g kg^−1^, 0.72 g kg^−1^, 5.06 mg kg^−1^, and 4.83 g kg^−1^, respectively (Table 1). The largest values of soil TN, TK, pH, SOM, TP, and AP were observed in the rhizosphere soil of *Tc*, while the lowest soil TN, TK, SOM, and TP values were observed in the rhizosphere soil of *Mj*, and the lowest pH and AP values were observed in the rhizosphere soil of *Pb* (Table 1). These soil chemical properties showed no marked variations among different dominant tree species in the natural *Tc* forest.

**Table 1:** The soil chemical properties of the dominant tree species in a natural *Toona ciliata* var. *pubescens* forest.

Soil chemical properties	*Tc*	*Pb*	*Mj*
TN (g kg^−1^)	6.45(±0.91)a	5.97(±0.87)a	5.34(±0.71)a
TK (g kg^−1^)	5.11(±0.64)a	4.95(±0.34)a	4.42(±0.60)a
pH	4.78(±0.25)a	4.32(±0.09)a	4.43(±0.08)a
SOM (g kg^−1^)	144.50(±21.49)a	132.88(±20.60)a	106.39(±12.88)a
TP (g kg^−1^)	0.81(±0.07)a	0.71(±0.04)a	0.64(±0.14)a
AP (mg kg^−1^)	6.82(±0.98)a	3.78(±0.92)a	4.58(±1.17)a

**Notes.**

Different letters in the same row indicate significant differences at *p* < 0.05, while the same letters indicate nonsignificant differences at *p* > 0.05 in the same row. Data are presented as the mean ± SE (*n* = 4). TN represents total nitrogen; TP represents total phosphorus; TK represents total potassium; SOM represents soil organic matter; AP represents available phosphorus; *Tc* represents *Toona ciliata* var. *pubescens*; *Pb* represents *Padus buergeriana*; *Mj* represents *Maesa japonica*.

### Overall pyrosequencing information

In the case of inter-soil and inter-root variations, the AMF sequence parameters did were nonsignificant among tree species (Table 2).

**Table 2:** AMF sequence information of dominant tree species in a natural *Toona ciliata* var. *pubescens* forest.

Tree species	Sample type	Total sequence number	Glomeromycota sequence number	Glomeromycota sequence proportion (%)
*Tc*	Soil	42826(±1425)a	31626(±1484)ab	74.15(±4.61)a
	Roots	45518 (±5028)a	30472(±55)b	69.58(±8.00)a
*Pb*	Soil	47684(±1143)a	33206(±2680)ab	69.56(±5.22)a
	Roots	49275(±5416)a	30304(±121)b	63.91(±7.36)a
*Mj*	Soil	47989(±3974)a	35273(±990)a	75.39(±7.65)a
	Roots	40866(±4127)a	30415(±61)b	76.87(±8.17)a

**Notes.**

Different letters indicate significant differences at *p* < 0.05, while the same letters indicate nonsignificant differences at *p* > 0.05 in the same column. Data are presented as the mean ±  SE (*n* = 4). *Tc*, represents *Toona ciliata* var. *pubescens*; *Pb* represents *Padus buergeriana*; *Mj* represents *Maesa japonica*.

Regarding soil-root variation (Table 2), the number of Glomeromycota sequences was 1.16-fold higher in rhizosphere soil than in roots for *Mj* (*p* <  0.05). The total AMF sequence number was higher in roots than in the rhizosphere soil in *Tc* and *Pb*, and the proportion of Glomeromycota sequences showed the opposite trend. Overall, the sequencing coverage and target sequence proportion could meet the experimental expectations by using the Illumina PE250 platform.

**Figure 2:**
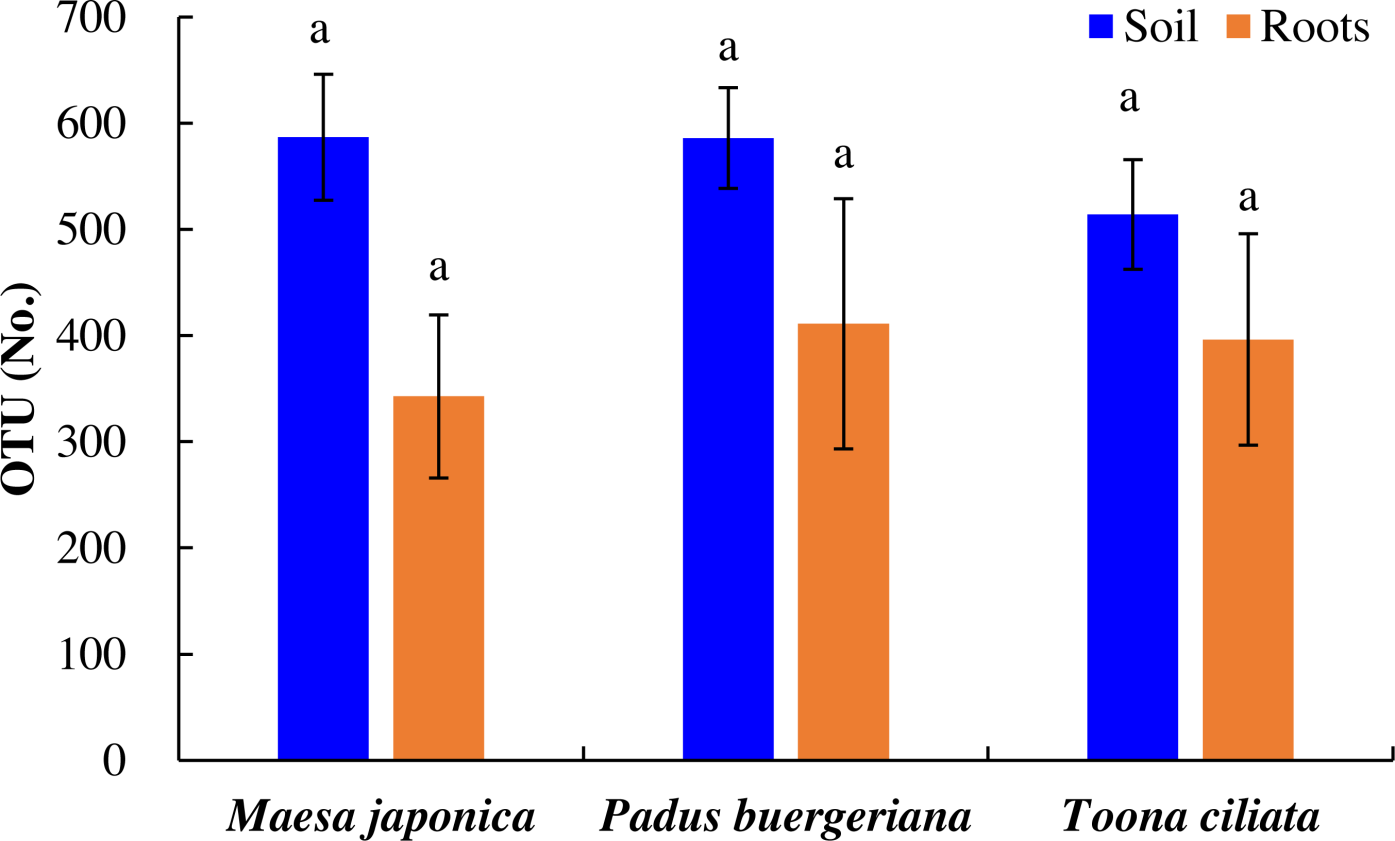
OTU variations in the roots and rhizosphere soil of dominant tree species in a natural *Toona ciliata* var. *pubescens* forest. Different letters indicate significant differences at *p* < 0.05, while the same letters indicate nonsignificant differences at *p* > 0.05. The error bar represents SE.

As shown in Fig. 2, the number of OTUs in *Tc*, *Mj*, and *Pb* rhizosphere soil was 514, 587, and 586, respectively, and OTU variations did were nonsignificant. Similarly, the number of OTUs in *Tc*, *Mj*, and *Pb* roots was 396, 343, and 411, respectively. In general, the number of OTUs was higher in the *Tc*, *Pb*, and *Mj* rhizosphere soil than in the corresponding roots (Fig. 2).

**Figure 3:**
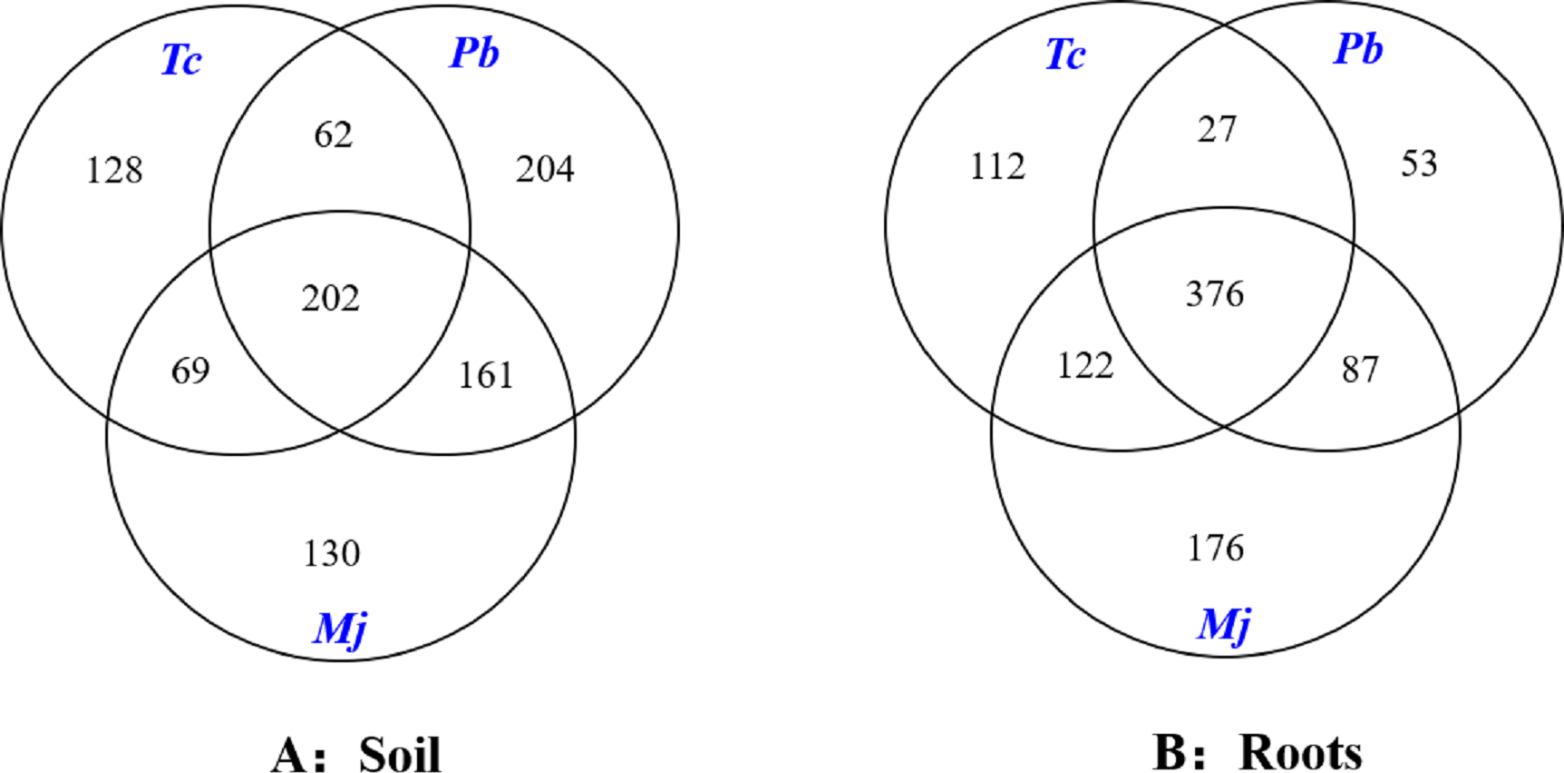
Venn diagram analysis of OTUs in dominant tree species in a natural *Toona ciliata* var. *pubescens* forest. *Tc* represents *T. ciliata* var. *pubescens*; *Pb* represents *Padus buergeriana*; *Mj* represents *Maesa japonica*.

The Venn diagram analysis results are shown in Fig. 3. A total of 202 mutual OTUs were found in the rhizosphere soil of these trees. The number of OTUs in *Pb* rhizosphere soil was the largest (204), followed by that in *Tc* (128) and *Mj* (130) rhizosphere soil. Mutual OTUs between *Tc* rhizosphere soil and *Mj* rhizosphere soil and between *Tc* rhizosphere soil and *Pb* rhizosphere soil were lower than those between *Pb* rhizosphere soil and *Mj* rhizosphere soil. In terms of roots (Fig. 3), a total of 376 mutual OTUs were found in the roots of these trees. The number of OTUs in *Tc* roots was the largest (112), followed by that in *Pb* (53) and *Mj* (76) roots. Mutual OTUs between *Tc* roots and *Mj* roots showed the highest value (122), while mutual OTUs between *Tc* roots and *Pb* roots showed the lowest value (27). Overall, we found that total mutual OTUs were higher in roots than in rhizosphere soil, while the number of unique OTUs showed the opposite trend for these trees.

### Composition of AMF communities

The composition of AMF communities was divided into nine components at the family level (Table 3). Glomeraceae was the most abundant in rhizosphere soil, and the relative abundance of Glomeraceae was 58.75%, 48.54%, and 63.92% in *Tc*, *Pb*, and *Mj* rhizosphere soil, respectively, and their differences were not significant. Subsequently, the relative abundances of unclassified fungi (16.01%), Ambisporaceae (16.86%), and Gigasporaceae (9.04%) were the second most abundant AMF in the *Tc*, *Pb*, and *Mj* rhizosphere soil, respectively. Compared with rhizosphere soil, the relative abundance of Glomeraceae in roots was more than 90% for these trees, as shown in Table 3. The relative abundance of Paraglomeraceae was the second most abundant AMF in *Tc* (2.98%) and *Pb* (2.74%) roots, while the relative abundance of Acaulosporaceae was the second most abundant AMF in *Mj* roots (2.80%).

**Table 3:** Variations in the relative abundance of the AMF community in dominant tree species in a natural *Toona ciliata var. pubescens* forest.

AMF community	*Tc*		*Pb*		*Mj*	
	Soil (%)	Roots (%)	Soil (%)	Roots (%)	Soil (%)	Roots (%)
Acaulosporaceae	2.29(±1.09)a	1.41(±0.60)a	0.66(±0.66)a	0.87(±0.33)a	3.28(±2.40)a	2.80(±1.53)a
Ambisporaceae	6.94(±6.29)a	0.17(±0.11)a	16.86(±12.83)a	0.22(±0.08)a	2.40(±0.89)a	0.20(±0.07)a
Archaeosporaceae	5.00(±1.45)ab	0.28(±0.05)c	8.15(±2.36)a	1.85(±0.57)bc	6.86(±1.97)a	1.02(±0.36)bc
Diversisporaceae	0.24(±0.24)a	0.00(±0.00)a	0.01(±0.01)a	0.00(±0.00)a	0.01(±0.00)a	0.03(±0.03)a
Geosiphonaceae	0.39(±0.37)a	0.08(±0.04)a	0.21(±0.04)a	0.05(±0.03)a	0.36(±0.26)a	0.17(±0.01)a
Gigasporaceae	6.44(±2.80)ab	0.28(±0.16)b	5.47(±3.58)ab	0.52(±0.08)b	9.04(±0.89)a	0.64(±0.16)b
Glomeraceae	58.75(±4.75)b	94.37(±2.04)a	48.54(±10.09)b	92.76(±1.47)a	63.92(±6.35)b	92.32(±1.00)a
Paraglomeraceae	3.93(±1.99)ab	2.98(±1.47)ab	8.28(±2.74)a	2.74(±0.76)ab	7.79(±2.25)ab	2.18(±0.50)b
Unclassified fungi	16.01(±3.94)a	0.68(±0.16)b	11.81(±7.12)ab	0.44(±0.18)b	6.32(±2.63)ab	0.62(±0.20)b

**Notes.**

Different letters indicate significant differences at *p* < 0.05, while the same letters indicate significant differences at *p* > 0.05 in the same row. Data are presented as the mean ± SE (*n* = 4). *Tc* represents *Toona ciliata var. pubescens*; *Pb* represents *Padus buergeriana*; *Mj* represents *Maesa japonica*.

Furthermore, the difference in AMF community composition between root and rhizosphere soil was also analysed in Table 3. There was no significant difference in the relative abundance of Acaulosporaceae, Ambisporaceae, Diversisporaceae, Geosiphonaceae, Paraglomeraceae, and unclassified fungi between root and rhizosphere soil. The relative abundance of Archaeosporaceae was significantly higher in rhizosphere soil than in roots (*p* < 0.05); in contrast, the relative abundance of Glomeraceae was significantly lower in rhizosphere soil than in roots in these trees (*p* <  0.05). The relative abundance of unclassified fungi was significantly lower in rhizosphere soil (0.68%) than in roots (16.01%) in *Tc* (*p* <  0.05). The relative abundance of Gigasporaceae was significantly higher in the rhizosphere soil than the roots in *Mj* (*p* <  0.05).

### Diversity of AMF communities

As shown in Table 4, the Chao index was lower in *Tc* rhizosphere soil than in *Pb* and *Mj* rhizosphere soil. Regarding inter-root variation, the Chao index was higher in *Tc* and *Mj* (>430) than in *Pb* (353.15). Moreover, the Chao index was significantly higher in the roots than in the rhizosphere soil in *Tc* and *Mj* (*p* < 0.05), while there was no significant difference in *Pb*.

**Table 4:** Variations in the diversity of AMF in dominant tree species in *Toona ciliata var. pubescens* natural forest.

Tree species	Sample type	Chao	Shannon	Simpson	Coverage
*Tc*	Soil	173.79(±47.58)c	3.62(±0.42)a	0.05 (±0.02)a	0.9995(±0.0001)a
	Root	431.79(±40.79)a	3.80(±0.28)a	0.07(±0.03)a	0.9986(±0.0003)b
*Pb*	Soil	266.73(±45.60)bc	3.48(±0.32)a	0.10(±0.06)a	0.9994(±0.0001)a
	Root	353.15(±5.43)ab	3.89(±0.08)a	0.04(±0.01)a	0.9987(±0.0002)b
*Mj*	Soil	247.91(±50.27)bc	3.91(±0.26)a	0.03(±0.01)a	0.9994(±0.0001)a
	Root	434.64(±24.56)a	4.11(±0.50)a	0.04(±0.01)a	0.9987(±0.0001)b

**Notes.**

Different letters indicate significant differences at *p* < 0.05, while the same letters indicate nonsignificant differences at *p* > 0.05 in the same column. Data are presented as the mean ± SE (*n* = 4). *Tc* represents *Toona ciliata var. pubescens*; *Pb* represents *Padus buergeriana*; *Mj* represents *Maesa japonica*.

For the Shannon and Simpson indexes, there were no significant inter-soil, inter-root or soil-root differences in the dominant tree species (Table 4).

In the case of the coverage index (Table 4), the average AMF community in the roots and rhizosphere soil of these trees was 0.9987 and 0.9994, respectively. Moreover, the coverage index was higher in rhizosphere soil than in roots for these trees (*p* < 0.05).

### Contribution of soil chemical properties to the differences in AMF community characteristics

**Figure 4:**
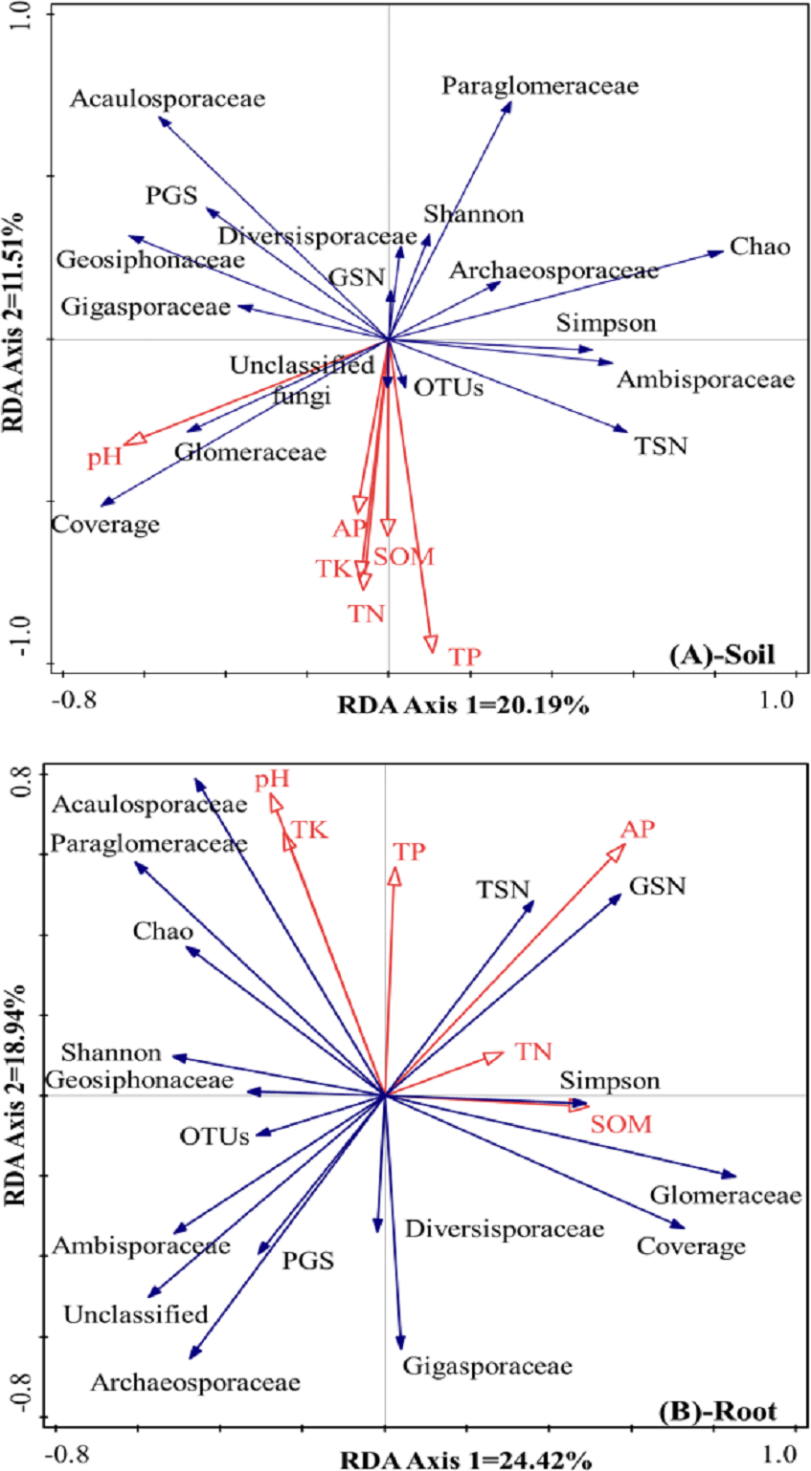
RDA results of the AMF community characteristics and soil chemical properties in a natural *Toona ciliata* var. *pubescens* forest (A: Soil, B: roots). TN represents total nitrogen; TP represents total phosphorus; TK represents total potassium; SOM represents soil organic matter; AP represents available phosphorus; TSN represents total sequence number; GSN represents Glomeromycota sequence number; PGS represents the proportion of Glomeromycota sequence; OTU represents operational taxonomic unit.

The differences in AMF composition and diversity were taken as response factors, and soil chemical properties were taken as explanatory factors. In rhizosphere soil (Fig. 4A), the first axis could explain 20.2% of the total differences in AMF characteristics, and the second axis could explain 11.5% of the total differences in AMF characteristics. Soil pH was positively correlated with the relative abundance of Glomeraceae and the coverage index but negatively correlated with the Chao index, the Simpson index, and the relative abundances of Archaeosporaceae and Ambisporaceae. SOM, TN, TK, TP, and AP showed positive correlations with OTUs and the relative abundance of unclassified fungi, and showed negative correlations with the Shannon index, the relative abundance of Diversisporaceae and Paraglomeraceae, and the Glomeromycota sequence number.

Regarding the roots, as shown in Fig. 4B, the first axis could explain 24.4% of the total differences in AMF characteristics, and the second axis could explain 18.9% of the total differences in AMF characteristics. Relatively large soil pH and TK and TP contents were usually accompanied by a relatively large relative abundance of Acaulosporaceae and a low relative abundance of Diversisporaceae and Gigasporaceae. AP was positively correlated with total sequence number and Glomeromycota sequence number but negatively correlated with the relative abundance of unclassified fungi, Ambisporaceae, Archaeosporaceae, and the proportion of Glomeromycota sequences. TN and SOM contents showed positive correlations with the Simpson index and the relative abundance of Glomeraceae and negative correlations with the Shannon index, OTUs, and the relative abundance of Geosiphonaceae.

## Discussion

### Illumina PE250 high-throughput sequencing method was used to study the characteristics of AMF community in a natural *Toona ciliata* var. *pubescens* forest

The Illumina PE250 high-throughput sequencing method was used to study the characteristics of the AMF community in a natural *Tc* forest. The total Glomeromycota sequence number was lower in *Tc* rhizosphere soil than in *Pb* and *Mj* rhizosphere soil, indicating that the Glomeromycota sequence was affected by the host plant. A previous study also showed that host trees could affect AMF spore communities in tropical forests (Lovelock, Andersen & Morton, 2003). The invasive plant species *Centaurea maculosa* altered AMF communities in the field (Mummey & Rillig, 2006). Moreover, the Glomeromycota sequence number was 1.04–1.60 times higher in the rhizosphere soil than in roots for *Tc*, *Pb*, and *Mj*, this finding indicated that rhizosphere soil of dominant tree species in the natural *Tc* forest could harbour a larger number of Glomeromycota sequences than roots.

In this study, 31626-35273 and 30304-30472 Glomeromycota sequences, forming 514-587 OTUs and 343-411 OTUs of the AMF community, were shown in the rhizosphere soil and roots, respectively, of dominant tree species in the natural *Tc* forest. Moreover, differences in OTUs between the roots and rhizosphere soil were observed, with the latter showing higher OTU values than the former. Many studies have revealed that rhizosphere soil-associated OTUs of the AMF community are higher than root-associated OTUs (Hu et al., 2019; Yang et al., 2013; Zheng et al., 2017). The AMF identification of root DNA ignored dormant spores and previously active symbionts (extracorporeal hyphae, dead plant root fragments, etc.) and therefore might underestimate the OTUs of root AMF during the measurement (Chen et al., 2014; Liu et al., 2015). Zheng et al. (2017) revealed that a minority of fungal OTUs were detected in both root and rhizosphere soil samples, and the degree to which these rhizosphere soil- and root-related fungal communities overlap may depend on local environmental conditions. AMF are asexual, obligately symbiotic fungi with unique morphologies and genomic structures and occupy the dual niche of the rhizosphere soil and the host roots (Vályi et al., 2016). Therefore, the combination of DNA extracted from both the roots and rhizosphere soil is suggested as an accurate method for identifying the AMF community (Chen et al., 2014; Liu et al., 2015).

### Characteristics of AMF diversity and community composition

The composition of the AMF community is thought to play a vital role in the establishment and succession of plant communities (O’Connor, Smith & Smith, 2002). An improved representation of the described Glomeromycota species in sequence databases would most likely increase the proportion of known species among these molecular taxa (Öpik et al., 2009). Among the nine components (Paraglomeraceae, Glomeraceae, Gigasporaceae, Geosiphonaceae, Diversisporaceae, Archaeosporaceae, Ambisporaceae, Acaulosporaceae, unclassified fungi), the composition of the AMF community in the rhizosphere soil and roots was mainly composed of Glomeraceae for all three tested tree species in the natural *Tc* forest (Table 3). Glomeraceae showed a high growth rate and rapid recovery after disturbance of spore production and hyphal networks (Radhika & Rodrigues, 2010), which might correspond to a ruderal life strategy. Moreover, the relative abundance of Glomeraceae in rhizosphere soil (48–64%) was significantly lower than that in the roots (>90%) of *Tc*, *Pb*, and *Mj*. Maherali & Klironomos (2007) have shown that the majority of fungal biomass in the Glomeraceae is found in the hyphae growing inside the root. These indicate a better ability of Glomeraceae to colonize plant roots when compared to that of the other AMF families. High colonization of roots but low colonization of rhizosphere soil by Glomeraceae has been previously described in cultures (Hart & Reader, 2002). Bonfim et al. (2016) found that although a high AMF diversity was determined in rhizosphere soil, only 14 AMF sequencing groups were detected inside the roots, and all had high similarity to Glomeraceae in the Atlantic Forest. In contrast, other families, such as Archaeosporaceae and Gigasporaceae, preferentially produce AMF extraradical hyphal biomass into rhizosphere soil compared to that produced in roots. These results were in agreement with earlier results showing that Archaeosporaceae and Gigasporaceae can be poor root colonizers (Hart & Reader, 2002; Varela-Cervero et al., 2015). Furthermore, more unclassified fungi were found in rhizosphere soil (6–17%) than in roots (0–1%), this finding is not surprising because *Tc* is a rare, local plant in Southeast China. Previous studies also identified several new species of Glomeromycota in the rhizospheres of endemic plants (Palenzuela et al., 2010; Varela-Cervero et al., 2015).

The diversity of AMF community is great significant for the maintenance and development of plant diversity (D’Souza & Rodrigues, 2013). AMF species diversity is one of the key portions of soil microbial diversity, which plays an important physiological and ecological role in promoting plant nutrient absorption, improving soil structure, and regulating the global carbon and nitrogen cycle (Ma et al., 2017). In this study, the Chao and Shannon indexes were used to analyze AMF community richness and diversity. The inter-soil or inter-root richness and diversity were not affected by different tree species. The larger the Chao and Shannon indexes were, the larger the AMF community richness and diversity. The Chao index of the AMF community was higher in roots than in the rhizosphere soil of dominant tree species in the natural *Tc* forest, suggesting that AMF community richness was greater in roots than in the rhizosphere soil. Here, we showed the efficiency of a natural *Tc* forest with respect to most of the AMF community richness being present in roots. However, the change in richness was actually the opposite of that of OTUs. The reason for this phenomenon was mainly due to AMF existing with a higher aggregation degree in roots than in rhizosphere soil, and there were more AMF groups with relatively low abundances in the rhizosphere soil, which correspondingly lowered the calculated richness index of the AMF community. This finding indicated that there were more concentrated AMF groups in the roots than in the rhizosphere soil, and these abundant and concentrated AMF groups might play an irreplaceable role in the long-term process of synergistic symbiosis within natural *Tc* forests. Similar result has been reported, suggesting higher AMF richness linked with plant roots than with soil samples (Mahmoudi et al., 2019).

### Relationships between soil chemical properties and the characteristics of AMF community

Changes in AMF community characteristics in different tree species are complex and depend on numerous abiotic and biotic factors. In particular, soil factors, such as soil type (Oehl et al., 2010), depth (Oehl et al., 2004), water content (Ji et al., 2007), pH (Bonfim et al., 2016), salt content (Guan et al., 2020), and fertility (Wang et al., 2009), have been shown to be important influencing factors since they are closely correlated with plant species and AMF community characteristics. In the current study, the influence of soil chemical properties on AMF community characteristics was analysed.

First, soil pH has been considered in many studies as an important environmental factor that shapes AMF community structure (Bonfim et al., 2013; Hu et al., 2019). These results indicated that the same soil pH played different roles in the variations in the AMF community characteristics in the roots and rhizosphere soil. Regarding rhizosphere soil, pH was positively correlated with the relative abundance of Glomeraceae and the coverage index but negatively correlated with the Chao index, the Simpson index, and the relative abundances of Archaeosporaceae and Ambisporaceae. However, a relatively high soil pH is usually accompanied by a large relative abundance of Acaulosporaceae and low relative abundances of Diversisporaceae and Gigasporaceae in roots. Soil pH directly affects the formation of AMF, as well as the spore production and genus distribution of AMF (Liu & Chen, 2007). Studies have indicated that the optimum pH of different AMF species is different; Glomus can adapt to a wide range of soil pH values but prefers to grow in alkaline and neutral soils, while *Acaulospora* prefers to grow in acidic soils (Gai & Liu, 2003). Zhang, Wang & Xing (1999) believed that adaptability to a relatively wide pH range is one of the key factors affecting the distribution range of a certain AMF species, and overly acidic or alkaline soil may have a negative effect on the survival and growth of some AMF species. In some cases, the effect of soil pH seems to be even more important than the host plant in the selection of AMF species (Bainard et al., 2014). The development of the AMF community can be selectively promoted by adjusting the soil pH value.

Second, soil nutrients mainly accounted for the changes in the AMF community characteristics between rhizosphere soil and roots (Fig. 4). Similar to pH, soil nutrients also exhibited different impacts on variations in AMF community characteristics in the rhizosphere soil and roots. For example, SOM, TN, TK, TP, and AP showed negative correlations with the Shannon index, the relative abundances of Diversisporaceae and Paraglomeraceae, and the Glomeromycota sequence number in rhizosphere soil; TP was usually accompanied by a large relative abundance of Acaulosporaceae and low relative abundances of Diversisporaceae and Gigasporaceae in roots. Among these nutrients, P is a relatively insoluble and immobile mineral, and AMF association is well considered to play important roles in host plant P uptake (Hu et al., 2019). Although previous studies have suggested that P fertilization reduces AMF colonization (Beauregard et al., 2010; Chen et al., 2014), the effects of P addition on AMF community characteristics are still under debate. For example, long-term (17 years) application of P fertilizer can significantly reduce the species richness, diversity index and mycelium density of AMF (Sheng et al., 2013). Hu et al. (2019) suggested that a decreased reliance of the host plant on AMF was found at a high P level. AP was significantly positively correlated with the AMF diversity index, species richness and evenness (Kou, Li & Xiao, 2019). Previous studies have also reported correlations between other soil nutrients and AMF community characteristics. In a certain range, the number of AMF spores increased with increasing organic matter content (1–2%), but beyond this range, the number of AMF spores decreased (Liu, Xu & Lv, 1999). Although there was a certain correlation with total carbon and TN, it was not significant (Kou, Li & Xiao, 2019). Available K was negatively correlated with observed species, the Chao richness index and Shannon’s diversity index, suggesting that it also played a key role in reducing AMF alpha diversity (Guan et al., 2020). Our results confirmed the results of previous study and provided new data to understand the dynamic role of AMF in the natural restoration of *Tc* forests.

## Conclusions

Similar inter-soil and inter-root AMF community characteristics between species were observed in this study. Glomeraceae was the most abundant AMF taxon of the roots and rhizosphere soil of the dominant tree species in the natural *Tc* forest. The relative abundance of Glomeraceae was significantly lower in rhizosphere soil than in the roots of the dominant tree species. The number of AMF community OTUs was higher in rhizosphere soil than in roots. However, the Chao index of the AMF community was higher in roots than in rhizosphere soil, showing the efficiency of natural *Tc* forests with respect to harbouring most of the AMF richness in roots. RDA determined that soil pH and nutrients impacted variations in AMF community characteristics in the roots and rhizosphere soil. These findings provide a better understanding of subtropical natural *Tc* forest regeneration, and a reference for ecological restoration and conservation of natural *Tc* forests from the perspective of underground microbial diversity.

##  Supplemental Information

10.7717/peerj.11331/supp-1 Supplemental Information 1 Raw data Soil chemical properties of the dominant tree species (analysis results are shown in Tables 1 and 4); the characteristics of the AMF community (pyrosequencing information, composition and diversity) in roots and rhizosphere soil associated with the dominant tree species (analysis results are shown in Tables 2–4 and Figs. 2–4).

## Additional Information and Declarations

### DNA Deposition

The following information was supplied regarding the deposition of DNA sequences: The sequences are available at GenBank: SRP277481, SAMN15815414 to SAMN15815425, SAMN15815492 to SAMN15815503.

### Data Availability

The following information was supplied regarding data availability: The raw data are available in the Supplementary File.

## References

[ref-1] Algoet PH, Cover TM (1988). A sandwich proof of the Shannon–McMillan–Breiman theorem. The Annals of Probability.

[ref-2] Bainard LD, Bainard JD, Hamel C, Gan YT (2014). Spatial and temporal structuring of arbuscular mycorrhizal communities is differentially influenced by abiotic factors and host crop in a semi-arid prairie agroecosystem. FEMS Microbiology Ecology.

[ref-3] Bao SD (2000). Soil and agricultural chemistry analysis.

[ref-4] Beauregard MS, Hamel C, Atul N, St-Arnaud M (2010). Long-term phosphorus fertilization impacts soil fungal and bacterial diversity but not AM fungal community in Alfalfa. Microbial Mcology.

[ref-5] Bonfim JA, Vasconcellos RLF, Gumiere T, Mescolotti DdLC, Oehl F, Cardoso EJBN (2016). Diversity of arbuscular mycorrhizal fungi in a Brazilian Atlantic forest toposequence. Microbial Ecology.

[ref-6] Bonfim JA, Vasconcellos RLF, Stürmer SL, Cardoso EJBN (2013). Arbuscular mycorrhizal fungi in the Brazilian Atlantic forest: a gradient of environmental restoration. Applied Soil Ecology.

[ref-7] Caravaca F, Alguacil MM, Barea JM, Roldán A (2005). Survival of inocula and native AM fungi species associated with shrubs in a degraded Mediterranean ecosystem. Soil Biology & Biochemistry.

[ref-8] Chao A (1984). Nonparametric-estimation of the number of classes in a population. Scandinavian Journal of Statistics.

[ref-9] Chen YL, Zhang X, Ye JS, Han HY, Wan SQ, Chen BD (2014). Six-year fertilization modifies the biodiversity of arbuscular mycorrhizal fungi in a temperate steppe in Inner Mongolia. Soil Biology and Biochemistry.

[ref-10] Dhar PP, Mridha MAU (2012). Biodiversity of arbuscular mycorrhizal associations in some forest trees of Aagoonia, Bangladesh. The Indian Forester.

[ref-11] D’Souza J, Rodrigues BF (2013). Biodiversity of Arbuscular Mycorrhizal (AM) fungi in mangroves of Goa in West India. Journal of Forestry Research.

[ref-12] Edgar RC (2013). UPARSE: highly accurate OTU sequences from microbial amplicon reads. Nature Methods.

[ref-13] Feng G, Li XL (2001). Arbuscular mycorrhizal ecology and physiology.

[ref-14] Fu FL, Zhang L, Yang QP, Liang YL, Tao D (2007). A study on the interspecific association of dominant species in Toona ciliates var. pubescens natural forests communities. Acta Agriculturae Universitatis Jiangxiensis.

[ref-15] Gai J, Liu R (2003). Effects of soil factors on arbuscular mycorrhizae (AM) fungi around roots of wild plants. Chinese Journal of Applied Ecology.

[ref-16] Gillespie AW, Farrell RE, Walley FL, Ross AR, Leinweber P, Eckhardt K-U, Regier TZ, Blyth RI (2011). Glomalin-related soil protein contains non-mycorrhizal-related heat-stable proteins, lipids and humic materials. Soil Biology and Biochemistry.

[ref-17] Guan B, Zhang HX, Wang XH, Yang SS, Chen M, Hou AX, Cagle GA, Han GX (2020). Salt is a main factor shaping community composition of arbuscular mycorrhizal fungi along a vegetation successional series in the Yellow River Delta. Catena.

[ref-18] Guo XY, Fu L, Zhang L, Su H, Liang YL (2017). Effects of forest soil and soil-borne fungi on seed germination and seedling survival of *Toona ciliata* var. *pubescens*. Forest Research.

[ref-19] Hart MM, Reader RJ (2002). Taxonomic basis for variation in the colonization strategy of arbuscular mycorrhizal fungi. New Phytologist.

[ref-20] Hoeksema JD, Chaudhary VB, Gehring CA, Johnson NC, Karst J, Koide RT, Pringle A, Zabinski C, Bever JD, Moore JC (2010). A meta-analysis of context-dependency in plant response to inoculation with mycorrhizal fungi. Ecology Letters.

[ref-21] Hu JL, Lin X, Bentivenga SP, Hou XY, Ji BM (2019). Intraradical and extraradical communities of AM fungi associated with alfalfa respond differently to long-term phosphorus fertilization. flora.

[ref-22] Huang HL, Zhang L, Guo XY, Liang YL, Liu ZG (2013). Fruiting characteristics and sexual fecundity of *Toona ciliata var. pubescens* populations in Jiulianshan national nature reserve. Scientia Silvae Sinicae.

[ref-23] Huang HL, Zhang L, Jia LM, Liang YL, Cai JH (2018). Fitness of sexual reproduction of *Toona ciliata var. pubescens* natural populations and their sexual reproduction and regeneration. Chinese Journal of Applied Ecology.

[ref-24] Huang HL, Zhang L, Liao CK (2012). Seed rain, soil seed bank, and natural regeneration of natural *Toona ciliata var. pubescens* forest. Chinese Journal of Applied Ecology.

[ref-25] Jeffries P, Gianinazzi S, Perotto S, Turnau K, Barea J-M (2003). The contribution of arbuscular mycorrhizal fungi in sustainable maintenance of plant health and soil fertility. Biology and Fertility of Soils.

[ref-26] Ji LL, Tan WF, Chen XH (2019). Arbuscular mycorrhizal mycelial networks and glomalin-related soil protein increase soil aggregation in Calcaric Regosol under well-watered and drought stress conditions. Soil & Tillage Research.

[ref-27] Ji CH, Zhang SB, Cai JP, Bai DS, Li XL, Feng G (2007). Arbuscular mycorrhizal fungal diversity in arid zones in northwestern China. Biodiversity Science.

[ref-28] Kou SM, Li SS, Xiao M (2019). Diversity of arbuscular mycorrhizal fungi in degraded pasture soils of Hulunbeier and its correlation with soil factors. Journal of Green Science Technology.

[ref-29] Kumar A, Sharma S, Mishra S (2010). Influence of arbuscular mycorrhizal (AM) fungi and salinity on seedling growth, solute accumulation, and mycorrhizal dependency of Jatropha curcas L. Journal of Plant Growth Regulation.

[ref-30] Liu RJ, Chen YL (2007). Mycorrhizology.

[ref-31] Liu J, Jiang JM, Chen YT (2014). Genetic diversity of central and peripheral populations of *Toona ciliata var. pubescens*, an endangered tree species endemic to China. Genetics and Molecular Research.

[ref-32] Liu J, Jiang JM, Zou J, Xu JL, Shen H, Diao SF (2013). Genetic diversity of central and peripheral populations of *Toona ciliata var. pubescens*, an endangered tree species endemic to China. Chinese Journal of Plant Ecology.

[ref-33] Liu YJ, Johnson NC, Mao L, Shi GX, Jiang SJ, Ma XJ, Du GZ, An LZ, Feng HY (2015). Phylogenetic structure of arbuscular mycorrhizal community shifts in response to increasing soil fertility. Soil Biology and Biochemistry.

[ref-34] Liu RJ, Li XL (2000). Arbuscular mycorrhiza and its application.

[ref-35] Liu RJ, Xu K, Lv ZF (1999). Ecological distribution of arbuscular mycorrhizal fungi in saline alkaline soils of China. Chinese Journal of Applied Ecology.

[ref-36] Lovelock CE, Andersen K, Morton JB (2003). Arbuscular mycorrhizal communities in tropical forests are affected by host tree species and environment. Oecologia.

[ref-37] Ma YX, Li GT, Liang TY, Ma Y, Pan YY, Tong CY (2017). Research progress of AM fungi species diversity and ecological function. Journal of Inner Mongolia Forestry Science Technology.

[ref-38] Maherali H, Klironomos JN (2007). Influence of phylogeny on fungal community assembly and ecosystem functioning. Science.

[ref-39] Mahmoudi N, Cruz C, Mahdhi M, Mars M, Caeiro MF (2019). Arbuscular mycorrhizal fungi in soil, roots and rhizosphere of Medicago truncatula: diversity and heterogeneity under semi-arid conditions. PeerJ.

[ref-40] Marques MC, Burslem DF (2015). Multiple stage recruitment limitation and density dependence effects in two tropical forests. Plant Ecology.

[ref-41] Mummey DL, Rillig MC (2006). The invasive plant species *Centaurea maculosa* alters arbuscular mycorrhizal fungal communities in the field. Plant and Soil.

[ref-42] Münzbergová Z, Herben T (2005). Seed, dispersal, microsite, habitat and recruitment limitation: identification of terms and concepts in studies of limitations. Oecologia.

[ref-43] O’Connor PJ, Smith SE, Smith FA (2002). Arbuscular mycorrhizas influence plant diversity and community structure in a semiarid herbland. New Phytologist.

[ref-44] Oehl F, Laczko E, Bogenrieder A, Stahr K, Bösch R, Heijden MVD, Sieverding E (2010). Soil type and land use intensity determine the composition of arbuscular mycorrhizal fungal communities. Soil Biology and Biochemistry.

[ref-45] Oehl F, Sieverding E, Ineichen K, Ris EA, Boller T, Wiemken A (2004). Community structure of arbuscular mycorrhizal fungi at different soil depths in extensively and intensively managed agroecosystems. New Phytologist.

[ref-46] Öpik M, Metsis M, Daniell TJ, Zobel M, Moora M (2009). Large-scale parallel 454 sequencing reveals host ecological group specificity of arbuscular mycorrhizal fungi in a boreonemoral forest. New Phytologist.

[ref-47] Palenzuela J, Barea J-M, Ferrol N, Azcón-Aguilar C, Oehl FB (2010). Entrophospora nevadensis, a new arbuscular mycorrhizal fungus from Sierra Nevada National Park (southeastern Spain). Mycologia.

[ref-48] Radhika KP, Rodrigues BF (2010). Arbuscular mycorrhizal fungal diversity in some commonly occurring medicinal plants of Western Ghats, Goa region. Journal of Forestry Research.

[ref-49] Sheng M, Lalande R, Hamel C, Ziadi N (2013). Effect of long-term tillage and mineral phosphorus fertilization on arbuscular mycorrhizal fungi in a humid continental zone of Eastern Canada. Plant and Soil.

[ref-50] Simpson EH (1949). Measurement of diversity. Nature.

[ref-51] Smith SE, Read DJ (2008). Mycorrhizal symbiosis.

[ref-52] Sun B, Zhang TL, Zhao QG (1995). Comprehensive evaluation of soil fertility in the hilly and mountainous region of Southeastern China. Acta Pedologica Sinica.

[ref-53] Thapar HS, Vijyan AK, Uniyal K (1992). Vesicular-arbuscular mycorrhizal associations and root colonization in some important tree species. The Indian Forester.

[ref-54] Vályi K, Mardhiah U, Rillig MC, Hempel S (2016). Community assembly and coexistence in communities of arbuscular mycorrhizal fungi. The Isme Journal.

[ref-55] Varela-Cervero S, Vasar M, Davison J, Barea JM, Öpik M, Azcón-Aguilar C (2015). The composition of arbuscular mycorrhizal fungal communities differs among the roots, spores and extraradical mycelia associated with five Mediterranean plant species. Environmental Microbiology.

[ref-56] Wang MY, Hu LB, Wang WH, Liu ST, Li M, Liu RJ (2009). Influence of long-term fixed fertilization on diversity of arbuscular mycorrhizal fungi. Pedosphere.

[ref-57] Yang GW, Liu N, Lu WJ, Wang S, Kan HM, Zhang YJ, Xu L, Chen YL (2014). The interaction between arbuscular mycorrhizal fungi and soil phosphorus availability influences plant community productivity and ecosystem stability. Journal of Ecology.

[ref-58] Yang W, Zheng Y, Gao C, He XH, Ding Q, Kim YC, Rui YC, Wang SP, Guo LD (2013). The arbuscular mycorrhizal fungal community response to warming and grazing differs between soil and roots on the Qinghai-Tibetan Plateau. PLOS ONE.

[ref-59] Yao XH, Yu ZP, Xiong Y, Yi LL, Tang M, Yang D, Yang QP (2017). Mian energy plant resources (NFBP) in Jiangxi Guanshan National Nature Reserve. South China Forestry Science.

[ref-60] Zhan X, Li P, Hui WK, Deng YW, Gan SM, Sun Y, Zhao XH, Chen XY, Deng XM (2019). Genetic diversity and population structure of Toona ciliata revealed by simple sequence repeat markers. Biotechnology and Biotechnological Equipment.

[ref-61] Zhang MQ, Wang YS, Xing LJ (1999). The relationship between the distribution of AM fungi and environmental factors. Mycosystema.

[ref-62] Zhang L, Zhang L, Lv LJ (2008). The influence of rehygroscopy-dehydration on seed vigor of *Toona ciliate var. pubescens*. Technology Development.

[ref-63] Zheng Y, Hu HW, Guo LD, Anderson LC, Powell JR (2017). Dryland forest management alters fungal community composition and decouples assembly of root- and soil-associated fungal communities. Soil Biology and Biochemistry.

